# GLP-1 receptor agonists—another promising therapy for Alport syndrome?

**DOI:** 10.1007/s44162-024-00065-8

**Published:** 2025-03-01

**Authors:** Jan Boeckhaus, Holly Mabillard, John A. Sayer

**Affiliations:** 1 Nephrology and Rheumatology, University Medical Center Göttingen, Göttingen, Germany; 2 Renal Services, Newcastle Upon Tyne Hospitals NHS Foundation Trust, Newcastle Upon Tyne, UK; 3 Translational and Clinical Research Institute, Faculty of Medical Sciences, Newcastle University, Central Parkway, Newcastle Upon Tyne, UK; 4 NIHR Newcastle Biomedical Research Centre, Newcastle Upon Tyne, UK; 5 Faculty of Medical Sciences, Biosciences Institute, Newcastle University, Central Parkway, Newcastle Upon Tyne, UK

**Keywords:** Alport syndrome (AS), Chronic kidney disease (CKD), Glucagon-like peptide-1 receptor agonists (GLP-1 RAs), Nephroprotection, Collagen IV, Genetic kidney disease

## Abstract

Alport syndrome (AS) is a progressive monogenic glomerular kidney disease characterised by kidney function decline, hearing loss, and ocular abnormalities, often leading to early-onset kidney failure (KF). While current therapies, such as renin-angiotensin system inhibitors (RASi), offer some benefits, many patients still experience KF at a young age, highlighting the need for additional treatment options. Glucagon-like peptide-1 receptor agonists (GLP-1 RAs) have emerged as promising agents with demonstrated cardiovascular and nephroprotective effects in type 2 diabetes (T2D) and chronic kidney disease (CKD) patients. Evidence from several major clinical trials has shown that GLP-1 RAs can reduce cardiovascular events and slow CKD progression by reducing albuminuria. Their potential mechanisms of action include anti-inflammatory, anti-fibrotic, and antioxidative effects, making them particularly relevant for the treatment of AS, where inflammation and fibrosis play crucial roles in disease progression. This review explores the therapeutic potential of GLP-1 RAs in AS, summarising pre-clinical and clinical data and elucidating the pathways through which GLP-1 RAs might offer renoprotective benefits. We advocate for further research into their application in AS and recommend the inclusion of AS patients in future clinical trials to better understand their impact on disease progression and patient outcomes.

## Introduction

For decades, patients with chronic kidney disease (CKD) had no pharmacological options to slow disease progression besides renin-angiotensin system (RASi) blockade, which were introduced into clinical practice in the early 1980s. In recent years, many new therapeutic options are being tested in clinical trials for patients with CKD. Alport syndrome (AS), a specific cause of progressive CKD, is the most common monogenetic glomerular kidney disease [1, 2]. Patients with AS can often develop kidney failure (KF) early in life. AS is characterised by glomerular proteinuria, progressive decline in kidney function, sensorineural hearing loss, and ocular lesions. Variants in the collagen IV genes (*COL4A3*, *COL4A4*, or *COL4A5*), encoding critical components of the glomerular basement membrane (GBM), cause Alport syndrome [3, 4]. These variants disrupt the structure and function of type IV collagen within the GBM, leading to impaired filtration and albuminuria, ultimately posing a high risk of KF [5]. Ocular lesions and progressive sensorineural hearing loss frequently accompany KF [6–9].

While RASi (angiotensin-converting–enzyme [ACE] inhibitors or angiotensin-receptor blockers [ARB]) demonstrably improved kidney function prognosis in AS, many patients still reach KF at a relatively young age [10–13]. This necessitates exploration of novel therapeutic options to delay the onset of KF.

The discovery of glucagon-like peptide-1 (GLP-1) receptor agonists has an intriguing biological origin linked to the venom of the Gila monster (*Heloderma suspectum*), a species of lizard native to the southwestern United States and Mexico [14]. The evolutionary advantage of GLP-1 in animals such as the Gila monster is thought to be linked to its role in metabolic regulation and energy efficiency, particularly in response to the harsh desert environment where prolonged fasting is common. GLP-1, through its enhancement of insulin secretion and delayed gastric emptying, allows the Gila monster to optimise nutrient absorption and regulate blood glucose levels during periods of food scarcity. Furthermore, the presence of exendin-4 in the lizard’s venom may serve to impair prey metabolism, potentially causing lethargy or hypoglycemia, thereby aiding in prey capture. This dual functionality underscores the adaptive value of GLP-1 in both metabolic management and survival in challenging ecological conditions [14]. Early research into the physiological functions of GLP-1 revealed its critical role in glucose metabolism, leading to its therapeutic potential in treating diabetes and obesity. However, it was in the saliva of the Gila monster that exendin-4, a GLP-1 receptor agonist, was identified. Exendin-4 mimics the effects of human GLP-1, but with greater stability and longer duration of action, laying the groundwork for the development of GLP-1 receptor-based drugs [15]. The identification of this peptide in lizard venom emphasises the diverse biochemical pathways evolutionarily adapted by organisms and highlights the translational potential of natural compounds in modern medicine.

Glucagon-like peptide-1 receptor agonists (GLP-1 RAs) are a class of medications mimicking the effects of GLP-1, a hormone regulating blood sugar levels. GLP-1 RAs have been shown to be effective in managing blood sugar in type 2 diabetes. Furthermore, increasing evidence suggests these medications positively influence cardiovascular risk in patients with and without diabetes. Additionally, they exhibit kidney protective effects in animal models of kidney disease and clinical trials (Table 1).

**Table 1:** Summary of trials using GLP-1 RAs

Trial	Year	Objective	Design	Population	Primary outcome	Results
SUSTAIN-6	2016	Investigate semaglutide’s effect on CVD risk in T2D patients	Randomized, double-blind, placebo-controlled	Patients aged 50 + with T2D, established CVD, heart failure, or CKD (stage III +), or aged 60 + with CVD risk factors	Composite of CV death, non-fatal MI, or non-fatal stroke	Semaglutide reduced the composite outcome significantly (6.6% vs. 8.9% in placebo; HR 0.74, CI 0.58–0.95; *p* < 0.001). Fewer serious adverse events occurred, but more patients discontinued due to gastrointestinal side effects
AMPLITUDE-O	2021	Investigate efpeglenatide’s effect on CV and CKD outcomes in T2D patients	Randomized, double-blind, placebo-controlled	Patients with T2D and history of CVD or eGFR 25.0–59.9 ml/min/1.73 m^2^	CV events and composite renal outcomes	Efpeglenatide reduced CV events and renal outcomes (13.0% vs. 18.4% in placebo). Gastrointestinal side effects were higher with efpeglenatide
REWIND	2019	Investigate dulaglutide’s effect on major CV events in T2D patients	Randomized, double-blind, placebo-controlled	Patients aged 50 + with T2D, with prior CV event or risk factors	First occurrence of non-fatal MI, non-fatal stroke, or CV death	Dulaglutide reduced major CV events (12.0% vs. 13.4% in placebo; HR 0.88, CI 0.79–0.99; *p* = 0.026). Gastrointestinal events more frequent with dulaglutide
Harmony Outcomes	2018	Investigate albiglutide’s effect on CV outcomes in T2D patients	Randomized, double-blind, placebo-controlled	Patients aged 40 + with T2D and established CVD	Composite of CV death, MI, or stroke	Albiglutide reduced CV events (7% vs. 9% in placebo; HR 0.78, CI 0.68–0.90; *p* < 0.0001)
LEADER	2016	Investigate liraglutide’s effect on CV outcomes in T2D patients	Randomized, double-blind, placebo-controlled	Patients with T2D and high CV risk	Composite of death from cardiovascular causes, non-fatal MI, or non-fatal stroke	Liraglutide reduced CV events (13.0% vs. 14.9% in placebo; HR 0.87, CI 0.78–0.97; *p* < 0.001 for non-inferiority; *p* = 0.01 for superiority)
SELECT	2023	Investigate semaglutide’s effect on CV outcomes in overweight/obese individuals without diabetes	Randomized, double-blind, placebo-controlled	Overweight/obese patients aged 45 + with established CVD	Composite of CV death, non-fatal MI, or non-fatal stroke	Semaglutide reduced CV events (6.5% vs. 8.0% in placebo; HR 0.80, CI 0.72–0.90; *p* < 0.001). More discontinuation in semaglutide group due to side effects
AWARD-PEDS	2022	Investigate dulaglutide’s efficacy and safety in adolescents with T2D	Randomized, double-blind, placebo-controlled	Adolescents aged 10–18 with BMI above the 85th percentile	Change in HbA1c	Dulaglutide reduced HbA1c levels significantly compared to placebo (− 0.6 to − 0.9%). Higher incidence of gastrointestinal adverse events in dulaglutide group
Flow study	2023	Investigate semaglutide’s effect on kidney outcomes in CKD patients with T2D	Randomized, double-blind, multinational phase 3b trial	Patients with CKD and T2D	Composite of eGFR decline, kidney failure, or death from kidney/CV causes	Semaglutide reduced primary outcome events by 24% (95% CI: 0.66–0.88; *p* = 0.0003). Slower eGFR decline observed, but more frequent discontinuation due to gastrointestinal side effects

Therefore, the 2022 KDIGO guidelines recommend long-acting GLP-1 RAs with established cardiovascular (CV) benefits as the preferred second-line therapy for glycemic control and CV risk reduction in adults with type 2 diabetes with established cardiovascular disease (CVD) or a high CV risk profile. This applies to patients who fail to achieve individualised glycemic targets despite receiving metformin and an SGLT2 inhibitor (SGLT2i) or who have a contraindication to these medications [16].

Some of the most well-known drugs include liraglutide, semaglutide, and dulaglutide. These drugs mimic the action of GLP-1 by stimulating insulin secretion, inhibiting glucagon release, and slowing gastric emptying, all of which contribute to improved glycemic control and weight loss. Other agents, such as exenatide and its extended-release version, are also commonly prescribed. These medications differ in their pharmacokinetics, dosing regimens, and delivery mechanisms, but they all offer significant benefits in managing hyperglycemia and reducing body weight in patients with metabolic disorders [17].

While GLP-1 receptor agonists (GLP-1 RAs) have demonstrated broad nephroprotective effects in diabetic kidney disease, the specific focus of this review is on their potential application in Alport syndrome (AS). AS, as a genetically distinct condition characterised by mutations in collagen IV genes, involves unique pathological mechanisms, including early-onset kidney fibrosis, oxidative stress, and inflammation, which GLP-1 RAs may address through their pleiotropic effects. This review provides a foundational overview of GLP-1 RAs to contextualise their mechanisms of action before exploring their targeted relevance in AS.

## GLP-1 receptor agonists—what does the evidence say?

GLP1-RAs are hypothesized to be a promising therapy for slowing the progression of diabetic CKD [18]. Some GLP-1 RAs (albiglutide, efpeglenatide, dulaglutide, liraglutide, and once-weekly semaglutide) reduce the risk of major adverse cardiovascular events (MACE) in people with type 2 diabetes (T2D) with established CVD or at high risk of CVD [19–23]. A summary of GLP-1 RA trials is featured in Table 1.

The SUSTAIN-6 trial investigated whether once-weekly semaglutide (0.5 mg or 1.0 mg) could reduce CVD risk in patients aged 50 or older with T2D. Participants had established CVD (including prior heart attack, stroke, or peripheral arterial disease), chronic heart failure or CKD (stage III or higher) or were aged 60 or older with at least one CVD risk factor. The primary outcome was the first occurrence of CV death, non-fatal myocardial infarction, or non-fatal stroke. The rate of the primary composite outcome was significantly lower in the semaglutide group (6.6%) compared to placebo (8.9%) (hazard ratio [HR], 0.74; 95% confidence interval [CI], 0.58 to 0.95; *p*< 0.001 for non-inferiority). While fewer serious adverse events occurred in the semaglutide group, more patients discontinued treatment due to side effects, primarily gastrointestinal issues [19].

The AMPLITUDE-O trial investigated the effect of efpeglenatide, an exendin-based GLP-1 RA, on CV and CKD outcomes in T2D patients. Patients with T2D and glycated hemoglobin > 7%, ≥ 18 years old with previous CVD, or ≥ 50 years old with an eGFR of 25–59.9 ml/min/1.73 m^2^, and one or more additional CVD risk factors were recruited. Participants were randomly assigned in a 1:1:1 ratio to receive weekly subcutaneous injections of efpeglenatide (4 mg or 6 mg) or placebo. Compared to placebo, efpeglenatide significantly reduced the risk of major adverse cardiovascular event. Additionally, a composite renal outcome event (kidney function decline or macroalbuminuria) occurred less frequently in the efpeglenatide groups (13.0%) compared to placebo (18.4%). However, efpeglenatide was associated with a higher incidence of gastrointestinal side effects such as diarrhoea and nausea compared to placebo [20].

The REWIND trial assessed the effect of the GLP-1 RA dulaglutide on major adverse CV events in patients with T2D, regardless of prior CVD. This multicenter, randomized, double-blind, placebo-controlled trial included patients aged at least 50 years with T2D who had either a previous CV event or risk factors. Participants were randomly assigned (1:1) to either subcutaneous injection of dulaglutide (1.5 mg/week) or placebo. The primary outcome was the first occurrence of non-fatal myocardial infarction, non-fatal stroke, or cardiovascular death. During a median follow-up of 5.4 years, the primary composite endpoint occurred in 12.0% of the dulaglutide group compared to 13.4% of the placebo group (HR 0.88, 95% CI 0.79–0.99; *p* = 0.026). Gastrointestinal adverse events were more frequent in the dulaglutide group (47.4%) compared to placebo (34.1%) (*p*< 0.0001) [21].

The Harmony Outcomes trial investigated the cardiovascular effects of once weekly albiglutide in patients aged 40 years with T2D and established CVD. This double-blind, randomized, placebo-controlled trial assigned participants to receive either a subcutaneous injection of albiglutide (30–50 mg) or placebo weekly, on top of their standard care. The primary outcome was the first occurrence of CV death, myocardial infarction, or stroke. The rate of the primary composite outcome was lower in the albiglutide group (7%) compared to placebo (9%). Albiglutide demonstrated both non-inferiority and superiority to placebo in reducing CV events (HR 0.78; 95% CI 0.68–0.90; *p*< 0.0001 for non-inferiority) [22].

The LEADER trial investigated whether liraglutide could reduce CV risk in patients with T2D and high CV risk. In this double-blind trial, participants were randomly assigned to receive liraglutide or placebo. The primary outcome, a composite of death from cardiovascular causes, non-fatal myocardial infarction, or non-fatal stroke, occurred significantly less frequently in the liraglutide group (13.0%) compared to placebo (14.9%) (HR, 0.87; 95% CI, 0.78 to 0.97; *p* < 0.001 for non-inferiority; *p*= 0.01 for superiority). Gastrointestinal events were the most common reason for discontinuing liraglutide [24].

The SELECT trial investigated whether semaglutide could reduce CV risk in overweight and obese individuals without diabetes. This multicenter, double-blind, randomized, placebo-controlled trial enrolled patients aged 45 or older with established CVD and a body mass index (BMI) of 27 or higher. Participants were randomly assigned to receive subcutaneous semaglutide (2.4 mg/week) or placebo. The primary endpoint, a composite of CV death, non-fatal myocardial infarction, or non-fatal stroke, occurred significantly less frequently in the semaglutide group (6.5%) compared to placebo (8.0%) (HR, 0.80; 95% CI, 0.72 to 0.90; *p*< 0.001). Notably, discontinuation due to adverse events was more common in the semaglutide group (16.6%) compared to placebo (8.2%) [25].

The AWARD-PEDS trial investigated the efficacy and safety of once-weekly dulaglutide in adolescents with T2D. Youths aged 10 to less than 18 years with a body mass index (BMI) above the 85th percentile were included. This double-blind, placebo-controlled trial employed a 1:1:1 randomization scheme. Participants received either lifestyle modifications, or a combination with metformin, with or without basal insulin. They were further randomized to receive once-weekly subcutaneous injections of placebo or dulaglutide (0.75 mg or 1.5 mg). The study included a 26-week open-label extension. At 26 weeks, mean glycated hemoglobin (HbA1c) levels increased in the placebo group (0.6%) while decreasing in the dulaglutide groups (− 0.6% in the 0.75 mg group and − 0.9% in the 1.5 mg group; both comparisons vs. placebo, *p*< 0.001). Dulaglutide therapy was associated with a higher incidence of gastrointestinal adverse events compared to placebo. The authors reported the safety profile of dulaglutide to be consistent with observations in adult populations [26].

Recently, the Flow study investigated the effects of once weekly semaglutide on kidney outcomes in patients with CKD and T2D. This randomized, double-blind, multinational, phase 3b trial included patients with an eGFR ≥ 50‒ ≤ 75 ml/min/1.73 m^2^ and urine albumin to creatinine ratio (UACR) > 300‒ < 5000 mg/g or eGFR ≥ 25‒ < 50 ml/min/1.73 m^2^and UACR > 100‒ < 5000 mg/g. Patients were randomized 1:1 to 1.0 mg once weekly semaglutide or placebo [27]. The composite primary endpoint was defined as KF (persistent eGFR < 15 ml/min/1.73 m^2^ or initiation of chronic kidney replacement therapy), persistent ≥ 50% reduction in eGFR or death from kidney-related or CV causes. An independent data and safety monitoring committee recommended early completion of the trial for efficacy. The reduction of primary-outcome events resulted in a 24% lower relative risk of the primary outcome in the semaglutide group (95% CI: 0.66–0.88; *p* = 0.0003, number needed to treat = 20, 95% CI 14 to 40). At 104 weeks, UACR was reduced by 12% in the placebo group and by 40% in the semaglutide group. While patients with semaglutide treatment had a slower eGFR decline (− 2.19 vs. − 3.36 ml/min/m^2^per year), it led to more frequent discontinuation mainly due to gastrointestinal side effects [28].

A pooled analysis of SUSTAIN 6 and LEADER trials demonstrated that semaglutide and liraglutide, significantly reduced albuminuria from baseline to 2 years after randomization by 24% versus placebo in patients with T2D [29]. This finding is particularly relevant because changes in albuminuria have been consistently linked to subsequent risk of KF [30]. A pre-specified analysis of SELECT trial, showed a clinically relevant reduction in albuminuria with a net treatment benefit of − 10.7% (95% CI − 13.2, − 8.2) in patients with albuminuria at baseline suggesting a beneficial kidney effect of once-weekly subcutaneous semaglutide 2.4 mg in patients with overweight/obesity and established CVD, without diabetes [31].

Consistent, a post hoc analysis of the LEADER trial investigated the association between changes in albuminuria within the first year and subsequent cardiovascular and renal events. This analysis demonstrated that patients who achieved a reduction in albuminuria within the first year experienced fewer cardiovascular and renal outcomes [32]. Moreover, UACR may have broader implications, potentially serving as a strong predictor of CV risk [33].

While the nephroprotective effects of GLP-1 RAs—such as reductions in albuminuria, oxidative stress, and inflammation—are well-documented in CKD, AS presents unique challenges and opportunities for these agents. The genetic basis of AS leads to structural and functional disruptions of the glomerular basement membrane (GBM), driving early-onset fibrosis and inflammation. These processes are amplified in AS compared to other glomerulopathies, making anti-fibrotic and antioxidative strategies like those offered by GLP-1 RAs particularly relevant. By targeting these shared but exacerbated pathways, GLP-1 RAs hold potential for modifying the disease trajectory in AS.

## Nephroprotection—what is the mechanism?

Studies that combined results from multiple clinical trials investigating CV outcomes suggest that GLP-1 RAs might also have nephroprotective potential in T2D. In particular, semaglutide, efpeglenatide, dulaglutide, and liraglutide were linked to a lower risk of developing combined kidney problems (macroalbuminuria, significant decline in kidney function, KF, and death from kidney disease). However, it is important to note that, until the FLOW trial, this benefit seemed mainly due to a reduced risk of persistently high level macroalbuminuria [34, 35].

The precise mechanisms by which GLP-1 RAs exert kidney-protective effects remain under investigation. While mediation analyses suggest that improved glycemic control, weight loss, and reduced blood pressure contribute to these benefits, they likely do not fully explain the observed renoprotection [36–38]. This implies involvement of additional, yet to be fully elucidated, mechanisms. Experimental studies to elucidate the beneficial effects of GLP-1 RA have mainly implicated the induction of natriuresis and the inhibition of oxidative stress, inflammation, and fibrosis [39–41]. In *ApoE*^−/−^ and *LDLr*^*−/−*^mice, GLP-1 RA significantly reduced arterial plaque formation, at least in part independent of weight or cholesterol changes. Studies on semaglutide further supported this mechanism by not only reversing aortic artery gene expression linked to atherosclerotic pathways in mice fed a Western diet, but also by decreasing plasma markers of systemic inflammation in an acute lipopolysaccharide model. Analysis of aortic atherosclerotic tissue revealed that multiple inflammatory pathways were downregulated by semaglutide, further solidifying its potential anti-inflammatory mechanism [42]. Correspondingly, GLP-1 receptor agonists have been shown to decrease inflammatory markers like C-reactive protein (CRP) and interleukin-6 (IL-6) [43, 44]. Advanced glycation end products (AGEs) accumulate with age, diabetes, and renal failure, promoting vascular inflammation through the AGE-RAGE axis. GLP-1 may mitigate this inflammation by downregulating RAGE expression via cyclic AMP signaling [45]. In addition, GLP-1 and its analogs have been shown to exert immunomodulatory effects by influencing macrophage polarization. While inflammatory M1 macrophages contribute to tissue injury and fibrosis, M2 macrophages exhibit anti-inflammatory properties and promote tissue repair and regeneration. By promoting an M2 phenotype, GLP-1 may contribute to reduced inflammation [46, 47]. Murine studies also showed that GLP-1 RA might be protective against renal oxidative stress by inhibition of NAD(P)H oxidase and by activation of the cAMP-protein kinase A pathway [48]. GLP-1 receptor expression in endothelial cells, vascular smooth muscle cells, macrophages, and monocytes suggests a potential anti-atherosclerotic effect of GLP-1 RAs [49–51]. Supporting this notion, GLP-1 RAs are reported to decrease reactive oxygen species production in endothelial cells, reduce circulating markers of inflammation, and slow atherosclerotic plaque formation by inhibiting the expression of adhesion molecules [43, 52]. Furthermore, GLP-1 RAs might promote a decreasing of endothelin levels, a potent vasoconstrictor, which is also involved in inflammatory and fibrotic processes [53, 54].

GLP-1 RAs have been shown to lower plasma angiotensin II levels, suggesting a link between the GLP-1 and RAAS pathways [55, 56]. This interaction may occur through two mechanisms: First, GLP-1 RA may decrease sodium reabsorption in the proximal tubule, leading to increased delivery of sodium chloride to the macula densa and subsequent activation of the tubuloglomerular feedback mechanism, which inhibits renin secretion [57]. Alternatively, GLP-1 RA might directly act on renin-producing cells within the juxtaglomerular apparatus, where GLP-1 receptors have been identified or might directly protect podocytes from apoptosis [58–60]. To further increase knowledge about the potential protective mechanism of GLP-1 RA, the mechanistic REMODEL trial (NCT04865770) is underway to further investigate these potential pathways [61].

Emerging evidence suggests a bidirectional relationship between leptin and GLP-1 signaling, with leptin potentially modulating GLP-1 release and action [62]. This interaction may influence inflammation, oxidative stress, and metabolic homeostasis, which are critical in CKD progression.

The mechanisms of kidney injury in AS, while overlapping with those in other CKD etiologies, are uniquely influenced by the collagen IV network’s structural integrity within the GBM. This distinguishes AS as a disease characterised by progressive glomerular sclerosis, tubulointerstitial fibrosis, and inflammation, driven by the downstream effects of GBM dysfunction. GLP-1 RAs, through their ability to inhibit oxidative stress and fibrosis while modulating inflammatory responses, could directly target these pathological processes. Unlike general CKD, where albuminuria is often the predominant driver of progression, the role of collagen IV mutations in AS creates a unique therapeutic target for GLP-1 RAs.

## What does this mean for Alport syndrome?

**Fig. 1:**
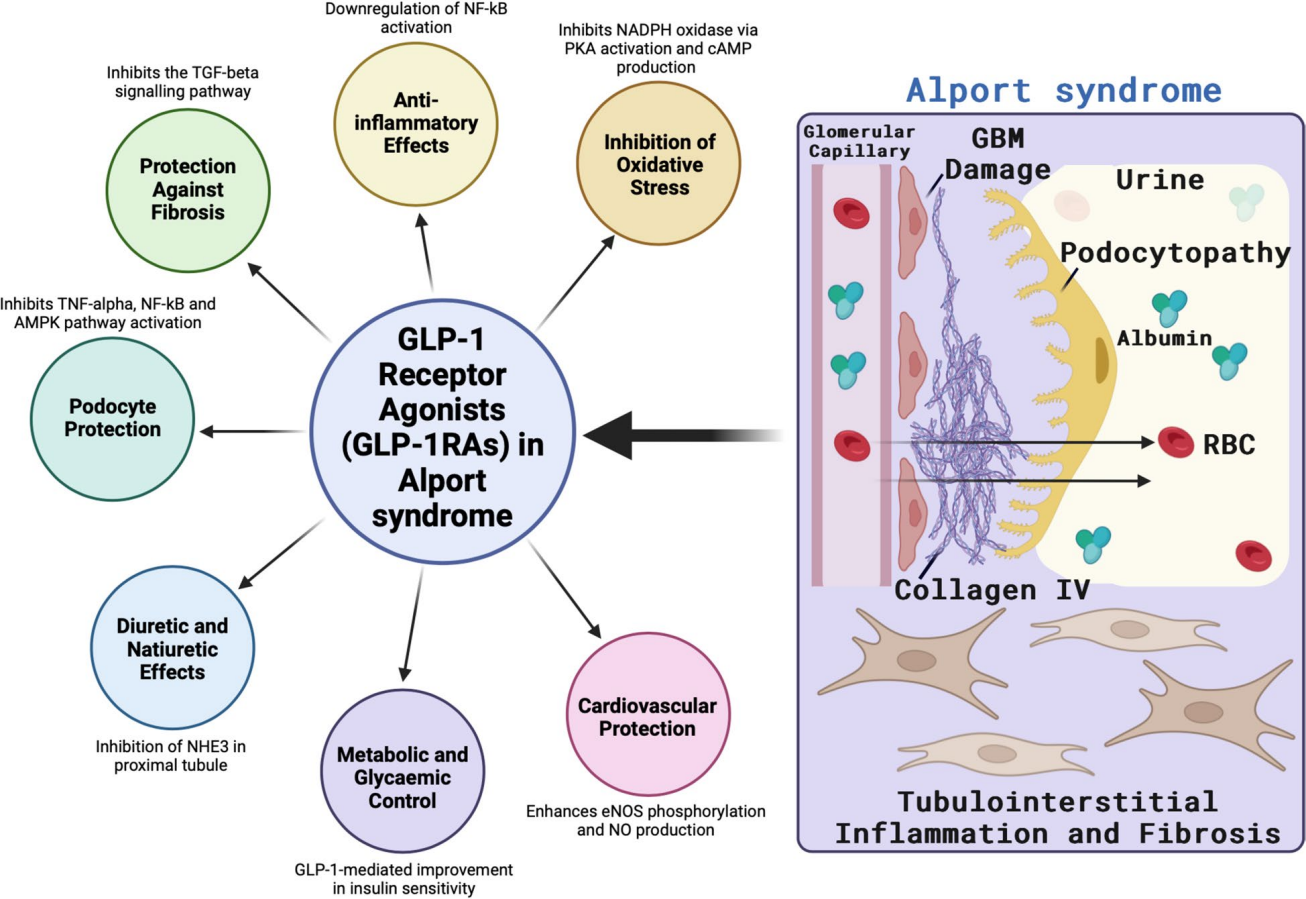
Potential protective mechanisms of GLP-1 RAs in Alport syndrome. Adapted from “Explaining Alport syndrome – lessons from the adult nephrology clinic” [66]. This figure summarises the key potential renoprotective effects of GLP-1 receptor agonists (GLP-1 RAs) in the context of Alport syndrome. Kidney-focused pathological sequelae in Alport syndrome include disruption of the collagen IV network which compromises the structural integrity of the glomerular basement membrane (GBM), subsequent albuminuria/proteinuria, hematuria, podocytopathy, progressive glomerular inflammation and sclerosis, tubulointerstitial inflammation, oxidative stress, and fibrosis. GLP-1 RAs inhibit oxidative stress by reducing NADPH oxidase activity through PKA activation and cAMP production, resulting in lower reactive oxygen species (ROS) levels in glomeruli and tubules [48]. GLP-1 RAs activate the Nrf2 signaling pathway to promote antioxidant defenses [67]. GLP-1 RAs inhibit fibrotic responses by suppressing the TGF-β pathway, reducing fibronectin and collagen deposition in the kidneys [68]. Podocyte protection is achieved through inhibition of TNF-α, NF-κB, and AMPK pathway activation, preserving glomerular filtration barrier integrity and reducing albuminuria [69]. Additionally, GLP-1 RAs promote diuresis and natriuresis by inhibiting the Na + /H + exchanger (NHE3) in proximal tubules, leading to enhanced sodium excretion and reduced fluid retention [57]. Red blood cell (RBC); glomerular basement membrane (GBM). Image created using BioRender

GLP-1 RAs now present a promising therapeutic approach for AS in a similar manner to other relatively recently discovered nephroprotective drugs, such as SGLT2 inhibitors and finerenone [63–65]. Potential protective mechanisms of GLP-1 RAs in AS are summarised in Fig. 1. Mechanistically, GLP-1 RAs exhibit renoprotective effects by reducing oxidative stress, inflammation, and fibrosis, processes central to the pathogenesis of AS [1, 3]. In addition to their anti-inflammatory properties, GLP-1 RAs might lower plasma angiotensin II levels, modulating the RAAS which is implicated in the progression of kidney disease [16, 34]. Clinical evidence from studies on diabetic CKD suggests that GLP-1 RAs not only improve glycemic control and reduce CV risk but also slow the progression of CKD through reduction in albuminuria, reduced systemic inflammation, enhanced endothelial function, and weight loss [37, 48]. Given the significant unmet need for novel therapeutic interventions in AS, particularly as many patients still progress to KF despite current treatments like RASi, GLP-1 RAs may offer an innovative strategy to delay KF in AS, potentially addressing both metabolic and inflammatory pathways involved in CKD progression [40, 42, 56].

Podocyte injury is a key contributor to glomerular dysfunction in Alport syndrome, driven by oxidative stress and inflammation. GLP-1 RAs have shown potential to protect podocytes by reducing TNF-α expression, inhibiting NF-κB activation, and preserving mitochondrial function, which collectively support glomerular barrier integrity [70]. Although direct effects of GLP-1 RAs on the GBM have not been fully elucidated, their anti-inflammatory, anti-fibrotic, and antioxidative properties may indirectly support GBM integrity. These mechanisms could help mitigate the structural and functional disruptions caused by collagen IV mutations in Alport syndrome.

Further research is warranted to elucidate the full therapeutic potential of GLP-1 RAs in AS but like many genetic kidney diseases, Alport syndrome patients have been excluded from important clinical trials exploring therapeutics that slow the progression of CKD. Including patients with Alport syndrome in general CKD treatment trials offers benefits such as larger sample sizes, cost-efficiency, and faster access to results, given shared disease mechanisms with many of the CKD etiologies. However, the heterogeneity of CKD may obscure disease-specific drug effects, underrepresent Alport patients, and delay the discovery of targeted therapies. Conversely, independent Alport syndrome trials provide tailored study designs and greater insights into disease-specific mechanisms, but face challenges with smaller sample sizes, higher costs, and longer trial durations. Thus, the choice between these approaches hinges on balancing the need for generalisibility with the specificity required for rare disease research.

Although the therapeutic benefits of GLP-1 RAs have been extensively studied in diabetic CKD, their application in AS requires further exploration. The genetic basis and early-onset nature of AS make it a distinct entity within CKD, with inflammation and fibrosis playing central roles from an early stage. GLP-1 RAs’ capacity to reduce inflammatory cytokines, promote podocyte health, and modulate oxidative stress pathways could have particular relevance in addressing the progressive decline seen in AS patients. This highlights the need for dedicated clinical trials to evaluate GLP-1 RAs in AS, where these pleiotropic mechanisms may have a large impact.

## Considering the risks

GLP-1 RA drugs, while generally considered effective and safe for the treatment of T2D and obesity, are associated with certain risks and side effects, as documented in the literature which must not be completely overlooked. Common adverse effects include gastrointestinal disturbances, such as nausea, vomiting, and diarrhoea, which often subside with continued use [19]. More serious, though less frequent, risks include pancreatitis and, in rare cases, pancreatic cancer, although the causal relationship remains debated [71]. Thyroid C-cell hyperplasia and an increased risk of medullary thyroid carcinoma have been observed in rodent studies, but these findings have not been definitively replicated in humans [72]. Other concerns include hypoglycemia, particularly when GLP-1 RA are used in combination with insulin or sulfonylureas, as well as potential CV effects [73]. Overall, while GLP-1 RA present several potential risks, the benefit-risk profile remains favorable in most patients when prescribed appropriately.

## Conclusion

The potential for GLP-1 RAs to serve as a therapeutic option in AS presents an exciting avenue for further exploration, particularly given the unmet need for treatments that slow the progression of kidney function decline in this patient population. The pleiotropic effects of GLP-1 RAs, including their anti-inflammatory, anti-fibrotic, and renoprotective mechanisms, hold promise for modifying disease progression in AS, which is driven by defects in collagen IV and associated inflammation, fibrosis, and oxidative stress in the glomerular basement membrane. Evidence from clinical trials in diabetic CKD suggests that GLP-1 RAs improve both cardiovascular and renal outcomes by reducing albuminuria and slowing the decline in kidney function. These beneficial effects, in addition to metabolic improvements in glycemic control and weight reduction, provide a compelling rationale for the further study of GLP-1 RAs in non-diabetic kidney diseases like AS. However, careful consideration must be given to the safety profile of GLP-1 RAs, particularly their gastrointestinal side effects and rare risks, such as pancreatitis and thyroid-related complications [19, 71]. Future research should focus on understanding the molecular mechanisms through which GLP-1 RAs exert their renoprotective effects in AS and other monogenic kidney diseases, with an emphasis on translating these findings into clinical practice. Further research is also needed to determine whether GLP-1 RAs have direct effects on the GBM, such as modulating collagen IV assembly or repair. Exploring these pathways could clarify their specific benefits in Alport syndrome compared to other forms of CKD. If proven effective, GLP-1 RAs could represent a novel and impactful treatment strategy for delaying or preventing kidney failure in patients with AS and other causes of CKD, thereby significantly improving their long-term prognosis and quality of life.

## Data Availability

No datasets were generated or analysed during the current study.

## References

[1] Hudson BG, Tryggvason K, Sundaramoorthy M, Neilson EG. Alport’s syndrome, Goodpasture’s syndrome, and type IV collagen. N Engl J Med. 2003;348(25):2543–56. 10.1056/NEJMra022296. 12815141 10.1056/NEJMra022296

[2] Groopman EE, Marasa M, Cameron-Christie S, et al. Diagnostic utility of exome sequencing for kidney disease. N England J Med. Published online December 26, 2018. 10.1056/NEJMoa1806891. 10.1056/NEJMoa1806891 PMC6510541 30586318

[3] Savige J, Storey H, Il Cheong H, et al. X-linked and autosomal recessive Alport syndrome: pathogenic variant features and further genotype-phenotype correlations. PLoS One. 2016;11(9):e0161802. 10.1371/journal.pone.0161802. 27627812 10.1371/journal.pone.0161802 PMC5023110

[4] Kashtan CE, Ding J, Garosi G, et al. Alport syndrome: a unified classification of genetic disorders of collagen IV α345: a position paper of the Alport Syndrome Classification Working Group. Kidney Int. 2018;93(5):1045–51. 10.1016/j.kint.2017.12.018. 29551517 10.1016/j.kint.2017.12.018

[5] Puapatanakul P, Miner JH. Alport syndrome and Alport kidney diseases – elucidating the disease spectrum. Curr Opin Nephrol Hypertens. 2024;33(3):283. 10.1097/MNH.0000000000000983. 38477333 10.1097/MNH.0000000000000983 PMC10990029

[6] Barker DF, Pruchno CJ, Jiang X, et al. A mutation causing Alport syndrome with tardive hearing loss is common in the western United States. Am J Hum Genet. 1996;58(6):1157–65. 8651292 PMC1915056

[7] Boeckhaus J, Strenzke N, Storz C, Gross O. Characterization of sensorineural hearing loss in children with Alport syndrome. Life (Basel). 2020;10(12):360. 10.3390/life10120360. 33352923 10.3390/life10120360 PMC7766141

[8] Kruegel J, Rubel D, Gross O. Alport syndrome—insights from basic and clinical research. Nat Rev Nephrol. 2013;9(3):170–8. 10.1038/nrneph.2012.259. 23165304 10.1038/nrneph.2012.259

[9] Kashtan CE. Alport syndromes: phenotypic heterogeneity of progressive hereditary nephritis. Pediatr Nephrol. 2000;14(6):502–12. 10.1007/s004670050804. 10872195 10.1007/s004670050804

[10] Gross O, Tönshoff B, Weber LT, et al. A multicenter, randomized, placebo-controlled, double-blind phase 3 trial with open-arm comparison indicates safety and efficacy of nephroprotective therapy with ramipril in children with Alport’s syndrome. Kidney Int. 2020;97(6):1275–86. 10.1016/j.kint.2019.12.015. 32299679 10.1016/j.kint.2019.12.015

[11] Boeckhaus J, Hoefele J, Riedhammer KM, et al. Lifelong effect of therapy in young patients with the COL4A5 Alport missense variant p.(Gly624Asp): a prospective cohort study. Nephrol Dial Transplant. Published online January 12, 2022:gfac006. 10.1093/ndt/gfac006. 10.1093/ndt/gfac006 35022790

[12] Zhang Y, Böckhaus J, Wang F, et al. Genotype–phenotype correlations and nephroprotective effects of RAAS inhibition in patients with autosomal recessive Alport syndrome. Pediatr Nephrol. 2021;36(9):2719–30. 10.1007/s00467-021-05040-9. 33772369 10.1007/s00467-021-05040-9 PMC8370956

[13] Gross O, Licht C, Anders HJ, et al. Early angiotensin-converting enzyme inhibition in Alport syndrome delays renal failure and improves life expectancy. Kidney Int. 2012;81(5):494–501. 10.1038/ki.2011.407. 22166847 10.1038/ki.2011.407

[14] Eng J, Kleinman WA, Singh L, Singh G, Raufman JP. Isolation and characterization of exendin-4, an exendin-3 analogue, from Heloderma suspectum venom. Further evidence for an exendin receptor on dispersed acini from guinea pig pancreas. J Biol Chem. 1992;267(11):7402–5. 10.1016/S0021-9258(18)42531-8. 1313797

[15] Baggio LL, Drucker DJ. Biology of incretins: GLP-1 and GIP. Gastroenterology. 2007;132(6):2131–57. 10.1053/j.gastro.2007.03.054. 17498508 10.1053/j.gastro.2007.03.054

[16] Rossing P, Caramori ML, Chan JCN, et al. KDIGO 2022 clinical practice guideline for diabetes management in chronic kidney disease. Kidney Int. 2022;102(5):S1–127. 10.1016/j.kint.2022.06.008. 36272764 10.1016/j.kint.2022.06.008

[17] Drucker DJ. Mechanisms of action and therapeutic application of glucagon-like peptide-1. Cell Metab. 2018;27(4):740–56. 10.1016/j.cmet.2018.03.001. 29617641 10.1016/j.cmet.2018.03.001

[18] Holliday MWJ, Frost L, Navaneethan SD. Emerging evidence for glucagon-like peptide-1 agonists in slowing chronic kidney disease progression. Curr Opin Nephrol Hypertens. 2024;33(3):331. 10.1097/MNH.0000000000000976. 38411162 10.1097/MNH.0000000000000976 PMC11126299

[19] Marso SP, Bain SC, Consoli A, et al. Semaglutide and cardiovascular outcomes in patients with type 2 diabetes. N Engl J Med. 2016;375(19):1834–44. 10.1056/NEJMoa1607141. 27633186 10.1056/NEJMoa1607141

[20] Gerstein HC, Sattar N, Rosenstock J, et al. Cardiovascular and renal outcomes with efpeglenatide in type 2 diabetes. N Engl J Med. 2021;385(10):896–907. 10.1056/NEJMoa2108269. 34215025 10.1056/NEJMoa2108269

[21] Gerstein HC, Colhoun HM, Dagenais GR, et al. Dulaglutide and cardiovascular outcomes in type 2 diabetes (REWIND): a double-blind, randomised placebo-controlled trial. Lancet. 2019;394(10193):121–30. 10.1016/S0140-6736(19)31149-3. 10.1016/S0140-6736(19)31149-3 31189511

[22] Hernandez AF, Green JB, Janmohamed S, et al. Albiglutide and cardiovascular outcomes in patients with type 2 diabetes and cardiovascular disease (Harmony Outcomes): a double-blind, randomised placebo-controlled trial. Lancet. 2018;392(10157):1519–29. 10.1016/S0140-6736(18)32261-X. 10.1016/S0140-6736(18)32261-X 30291013

[23] Kosiborod MN, Petrie MC, Borlaug BA, et al. Semaglutide in patients with obesity-related heart failure and type 2 diabetes. N Engl J Med. 2024;390(15):1394–407. 10.1056/NEJMoa2313917. 38587233 10.1056/NEJMoa2313917

[24] Marso SP, Daniels GH, Brown-Frandsen K, et al. Liraglutide and cardiovascular outcomes in type 2 diabetes. N Engl J Med. 2016;375(4):311–22. 10.1056/NEJMoa1603827. 27295427 10.1056/NEJMoa1603827 PMC4985288

[25] Lincoff AM, Brown-Frandsen K, Colhoun HM, et al. Semaglutide and cardiovascular outcomes in obesity without diabetes. N Engl J Med. 2023;389(24):2221–32. 10.1056/NEJMoa2307563. 37952131 10.1056/NEJMoa2307563

[26] Arslanian SA, Hannon T, Zeitler P, et al. Once-weekly dulaglutide for the treatment of youths with type 2 diabetes. N Engl J Med. Published online August 4, 2022. 10.1056/NEJMoa2204601. 10.1056/NEJMoa2204601 35658022

[27] Rossing P, Baeres FMM, Bakris G, et al. The rationale, design and baseline data of FLOW, a kidney outcomes trial with once-weekly semaglutide in people with type 2 diabetes and chronic kidney disease. Nephrol Dial Transplant. 2023;38(9):2041–51. 10.1093/ndt/gfad009. 36651820 10.1093/ndt/gfad009 PMC10469096

[28] Perkovic V, Tuttle KR, Rossing P, et al. Effects of semaglutide on chronic kidney disease in patients with type 2 diabetes. N Engl J Med. 2024;391(2):109–21. 10.1056/NEJMoa2403347. 38785209 10.1056/NEJMoa2403347

[29] Shaman AM, Bain SC, Bakris GL, et al. Effect of the glucagon-like peptide-1 receptor agonists semaglutide and liraglutide on kidney outcomes in patients with type 2 diabetes: pooled analysis of SUSTAIN 6 and LEADER. Circulation. 2022;145(8):575–85. 10.1161/CIRCULATIONAHA.121.055459. 34903039 10.1161/CIRCULATIONAHA.121.055459 PMC8860212

[30] Coresh J, Heerspink HJL, Sang Y, et al. Change in albuminuria and subsequent risk of end-stage kidney disease: an individual participant-level consortium meta-analysis of observational studies. Lancet Diabetes Endocrinol. 2019;7(2):115–27. 10.1016/S2213-8587(18)30313-9. 30635225 10.1016/S2213-8587(18)30313-9 PMC6379893

[31] Colhoun HM, Lingvay I, Brown PM, et al. Long-term kidney outcomes of semaglutide in obesity and cardiovascular disease in the SELECT trial. Nat Med. Published online May 25, 2024:1–9. 10.1038/s41591-024-03015-5. 10.1038/s41591-024-03015-5 PMC11271413 38796653

[32] Persson F, Bain SC, Mosenzon O, et al. Changes in albuminuria predict cardiovascular and renal outcomes in type 2 diabetes: a post hoc analysis of the LEADER trial. Diabetes Care. 2021;44(4):1020–6. 10.2337/dc20-1622. 33504496 10.2337/dc20-1622 PMC7985419

[33] Matsushita K, Coresh J, Sang Y, et al. Estimated glomerular filtration rate and albuminuria for prediction of cardiovascular outcomes: a collaborative meta-analysis of individual participant data. Lancet Diabetes Endocrinol. 2015;3(7):514–25. 10.1016/S2213-8587(15)00040-6. 26028594 10.1016/S2213-8587(15)00040-6 PMC4594193

[34] Sattar N, Lee MMY, Kristensen SL, et al. Cardiovascular, mortality, and kidney outcomes with GLP-1 receptor agonists in patients with type 2 diabetes: a systematic review and meta-analysis of randomised trials. Lancet Diabetes Endocrinol. 2021;9(10):653–62. 10.1016/S2213-8587(21)00203-5. 34425083 10.1016/S2213-8587(21)00203-5

[35] Kristensen SL, Rørth R, Jhund PS, et al. Cardiovascular, mortality, and kidney outcomes with GLP-1 receptor agonists in patients with type 2 diabetes: a systematic review and meta-analysis of cardiovascular outcome trials. Lancet Diabetes Endocrinol. 2019;7(10):776–85. 10.1016/S2213-8587(19)30249-9. 31422062 10.1016/S2213-8587(19)30249-9

[36] Mann JFE, Muskiet MHA. Incretin-based drugs and the kidney in type 2 diabetes: choosing between DPP-4 inhibitors and GLP-1 receptor agonists. Kidney Int. 2021;99(2):314–8. 10.1016/j.kint.2020.08.036. 33509353 10.1016/j.kint.2020.08.036

[37] Mann JFE, Buse JB, Idorn T, et al. Potential kidney protection with liraglutide and semaglutide: exploratory mediation analysis. Diabetes Obes Metab. 2021;23(9):2058–66. 10.1111/dom.14443. 34009708 10.1111/dom.14443 PMC8453827

[38] Buse JB, Bain SC, Mann JFE, et al. Cardiovascular risk reduction with liraglutide: an exploratory mediation analysis of the LEADER trial. Diabetes Care. 2020;43(7):1546–52. 10.2337/dc19-2251. 32366578 10.2337/dc19-2251 PMC7305014

[39] Alicic RZ, Cox EJ, Neumiller JJ, Tuttle KR. Incretin drugs in diabetic kidney disease: biological mechanisms and clinical evidence. Nat Rev Nephrol. 2021;17(4):227–44. 10.1038/s41581-020-00367-2. 33219281 10.1038/s41581-020-00367-2

[40] Gallo G, Volpe M. Potential mechanisms of the protective effects of the cardiometabolic drugs type-2 sodium–glucose transporter inhibitors and glucagon-like peptide-1 receptor agonists in heart failure. Int J Mol Sci. 2024;25(5):2484. 10.3390/ijms25052484. 38473732 10.3390/ijms25052484 PMC10931718

[41] Kawanami D, Takashi Y. GLP-1 receptor agonists in diabetic kidney disease: from clinical outcomes to mechanisms. Front Pharmacol. 2020;11:967. 10.3389/fphar.2020.00967. 32694999 10.3389/fphar.2020.00967 PMC7338581

[42] Rakipovski G, Rolin B, Nøhr J, et al. The GLP-1 analogs liraglutide and semaglutide reduce atherosclerosis in ApoE−/− and LDLr−/− mice by a mechanism that includes inflammatory pathways. JACC Basic Transl Sci. 2018;3(6):844–57. 10.1016/j.jacbts.2018.09.004. 30623143 10.1016/j.jacbts.2018.09.004 PMC6314963

[43] Luna-Marco C, de Marañon AM, Hermo-Argibay A, et al. Effects of GLP-1 receptor agonists on mitochondrial function, inflammatory markers and leukocyte-endothelium interactions in type 2 diabetes. Redox Biol. 2023;66: 102849. 10.1016/j.redox.2023.102849. 37591012 10.1016/j.redox.2023.102849 PMC10457591

[44] Mazidi M, Karimi E, Rezaie P, Ferns GA. Treatment with GLP1 receptor agonists reduce serum CRP concentrations in patients with type 2 diabetes mellitus: a systematic review and meta-analysis of randomized controlled trials. J Diabetes Complications. 2017;31(7):1237–42. 10.1016/j.jdiacomp.2016.05.022. 28479155 10.1016/j.jdiacomp.2016.05.022

[45] Ishibashi Y, Matsui T, Takeuchi M, Yamagishi SI. Glucagon-like peptide-1 (GLP-1) inhibits advanced glycation end product (AGE)-induced up-regulation of VCAM-1 mRNA levels in endothelial cells by suppressing AGE receptor (RAGE) expression. Biochem Biophys Res Commun. 2010;391(3):1405–8. 10.1016/j.bbrc.2009.12.075. 20026306 10.1016/j.bbrc.2009.12.075

[46] Shiraishi D, Fujiwara Y, Komohara Y, Mizuta H, Takeya M. Glucagon-like peptide-1 (GLP-1) induces M2 polarization of human macrophages via STAT3 activation. Biochem Biophys Res Commun. 2012;425(2):304–8. 10.1016/j.bbrc.2012.07.086. 22842565 10.1016/j.bbrc.2012.07.086

[47] Chen J, Mei A, Wei Y, et al. GLP-1 receptor agonist as a modulator of innate immunity. Front Immunol. 2022;13:997578. 10.3389/fimmu.2022.997578. 36569936 10.3389/fimmu.2022.997578 PMC9772276

[48] Fujita H, Morii T, Fujishima H, et al. The protective roles of GLP-1R signaling in diabetic nephropathy: possible mechanism and therapeutic potential. Kidney Int. 2014;85(3):579–89. 10.1038/ki.2013.427. 24152968 10.1038/ki.2013.427

[49] Marx N, Husain M, Lehrke M, Verma S, Sattar N. GLP-1 receptor agonists for the reduction of atherosclerotic cardiovascular risk in patients with type 2 diabetes. Circulation. 2022;146(24):1882–94. 10.1161/CIRCULATIONAHA.122.059595. 36508493 10.1161/CIRCULATIONAHA.122.059595

[50] Pahud de Mortanges A, Sinaci E, Salvador D, et al. GLP-1 receptor agonists and coronary arteries: from mechanisms to events. Front Pharmacol. 2022;13:856111. 10.3389/fphar.2022.856111. 35370744 10.3389/fphar.2022.856111 PMC8964343

[51] Song X, Jia H, Jiang Y, et al. Anti-atherosclerotic effects of the glucagon-like peptide-1 (GLP-1) based therapies in patients with type 2 diabetes mellitus: a meta-analysis. Sci Rep. 2015;5(1):10202. 10.1038/srep10202. 26111974 10.1038/srep10202 PMC4481643

[52] Alharby H, Abdelati T, Rizk M, et al. Association of fasting glucagon-like peptide-1 with oxidative stress and subclinical atherosclerosis in type 2 diabetes. Diabetes Metab Syndr. 2019;13(2):1077–80. 10.1016/j.dsx.2019.01.031. 31336447 10.1016/j.dsx.2019.01.031

[53] Kowalczyk A, Kleniewska P, Kolodziejczyk M, Skibska B, Goraca A. The role of endothelin-1 and endothelin receptor antagonists in inflammatory response and sepsis. Arch Immunol Ther Exp. 2015;63(1):41–52. 10.1007/s00005-014-0310-1. 10.1007/s00005-014-0310-1 PMC4289534 25288367

[54] Dai Y, Mehta JL, Chen M. Glucagon-like peptide-1 receptor agonist liraglutide inhibits endothelin-1 in endothelial cell by repressing nuclear factor-kappa B activation. Cardiovasc Drugs Ther. 2013;27(5):371–80. 10.1007/s10557-013-6463-z. 23657563 10.1007/s10557-013-6463-z

[55] Skov J, Dejgaard A, Frøkiær J, et al. Glucagon-like peptide-1 (GLP-1): effect on kidney hemodynamics and renin-angiotensin-aldosterone system in healthy men. J Clin Endocrinol Metab. 2013;98(4):E664–71. 10.1210/jc.2012-3855. 23463656 10.1210/jc.2012-3855

[56] Skov J, Persson F, Frøkiær J, Christiansen JS. Tissue renin–angiotensin systems: a unifying hypothesis of metabolic disease. Front Endocrinol. 2014;5:23. 10.3389/fendo.2014.00023. 10.3389/fendo.2014.00023 PMC3938116 24592256

[57] Gutzwiller JP, Tschopp S, Bock A, et al. Glucagon-like peptide 1 induces natriuresis in healthy subjects and in insulin-resistant obese men. J Clin Endocrinol Metab. 2004;89(6):3055–61. 10.1210/jc.2003-031403. 15181098 10.1210/jc.2003-031403

[58] Wang J, Zhou Y, Long D, Wu Y, Liu F. GLP-1 receptor agonist, liraglutide, protects podocytes from apoptosis in diabetic nephropathy by promoting white fat browning. Biochem Biophys Res Commun. 2023;664:142–51. 10.1016/j.bbrc.2023.04.012. 37167707 10.1016/j.bbrc.2023.04.012

[59] Asmar A, Simonsen L, Asmar M, et al. Renal extraction and acute effects of glucagon-like peptide-1 on central and renal hemodynamics in healthy men. American Journal of Physiology-Endocrinology and Metabolism. 2015;308(8):E641–9. 10.1152/ajpendo.00429.2014. 25670826 10.1152/ajpendo.00429.2014

[60] Pyke C, Heller RS, Kirk RK, et al. GLP-1 receptor localization in monkey and human tissue: novel distribution revealed with extensively validated monoclonal antibody. Endocrinology. 2014;155(4):1280–90. 10.1210/en.2013-1934. 24467746 10.1210/en.2013-1934

[61] Bjornstad P, Cherney D, Lawson J, et al. MO399: remodel: a mechanistic trial evaluating the effects of semaglutide on the kidneys in people with type 2 diabetes and chronic kidney disease. Nephrol Dial Transplant. 2022;37(Supplement_3):gfac070.013. 10.1093/ndt/gfac070.013.

[62] Barakat GM, Ramadan W, Assi G, Khoury NBE. Satiety: a gut–brain–relationship. J Physiol Sci. 2024;74(1):11. 10.1186/s12576-024-00904-9. 38368346 10.1186/s12576-024-00904-9 PMC10874559

[63] Gross O, Boeckhaus J, Weber LT, et al. Protocol and rationale for a randomized controlled SGLT2 inhibitor trial in pediatric and young adult populations with chronic kidney disease: DOUBLE PRO-TECT Alport. Nephrol Dial Transplant. Published online August 9, 2024:gfae180. 10.1093/ndt/gfae180. 10.1093/ndt/gfae180 PMC11960741 39122650

[64] Pearce H, Mabillard H. Finerenone and other future therapeutic options for Alport syndrome. J Rare Dis. 2023;2(1):18. 10.1007/s44162-023-00022-x. 10.1007/s44162-023-00022-x PMC11489166 39429698

[65] Mabillard H, Sayer JA. SGLT2 inhibitors – a potential treatment for Alport syndrome. Clin Sci. 2020;134(4):379–88. 10.1042/CS20191276. 10.1042/CS20191276 32064497

[66] Mabillard H, Ryan R, Tzoumas N, Gear S, Sayer JA. Explaining Alport syndrome—lessons from the adult nephrology clinic. J Rare Dis. 2024;3(1):14. 10.1007/s44162-024-00036-z. 10.1007/s44162-024-00036-z PMC11088994 38745975

[67] Oh YS, Jun HS. Effects of glucagon-like peptide-1 on oxidative stress and Nrf2 signaling. Int J Mol Sci. 2018;19(1):26. 10.3390/ijms19010026. 10.3390/ijms19010026 PMC5795977 29271910

[68] Kodera R, Shikata K, Kataoka HU, et al. Glucagon-like peptide-1 receptor agonist ameliorates renal injury through its anti-inflammatory action without lowering blood glucose level in a rat model of type 1 diabetes. Diabetologia. 2011;54(4):965–78. 10.1007/s00125-010-2028-x. 21253697 10.1007/s00125-010-2028-x

[69] Barutta F, Bellini S, Gruden G. Mechanisms of podocyte injury and implications for diabetic nephropathy. Clin Sci. 2022;136(7):493–520. 10.1042/CS20210625. 10.1042/CS20210625 PMC9008595 35415751

[70] Liu J, Guo S, Li H, Liu XY. Effects of glucagon-like peptide-1 receptor agonists (GLP-1RAs) on podocytes, inflammation, and oxidative stress in patients with diabetic nephropathy (DN). Pak J Med Sci. 2022;38(5):1170. 10.12669/pjms.38.5.4719. 35799717 10.12669/pjms.38.5.4719 PMC9247776

[71] Butler PC, Elashoff M, Elashoff R, Gale EAM. A critical analysis of the clinical use of incretin-based therapies: are the GLP-1 therapies safe? Diabetes Care. 2013;36(7):2118–25. 10.2337/dc12-2713. 23645885 10.2337/dc12-2713 PMC3687282

[72] Drucker DJ, Sherman SI, Gorelick FS, Bergenstal RM, Sherwin RS, Buse JB. Incretin-based therapies for the treatment of type 2 diabetes: evaluation of the risks and benefits. Diabetes Care. 2010;33(2):428–33. 10.2337/dc09-1499. 20103558 10.2337/dc09-1499 PMC2809297

[73] Nauck MA, Meier JJ. Incretin hormones: their role in health and disease. Diabetes Obes Metab. 2018;20(S1):5–21. 10.1111/dom.13129. 29364588 10.1111/dom.13129

